# LPS/TLR4-mediated stromal cells acquire an invasive phenotype and are implicated in the pathogenesis of adenomyosis

**DOI:** 10.1038/srep21416

**Published:** 2016-02-22

**Authors:** Jing Guo, Li Chen, Ning Luo, Caixia Li, Rong Chen, Xiaoyan Qu, Mingmin Liu, Le Kang, Zhongping Cheng

**Affiliations:** 1 Department of Obstetrics and Gynecology, Yangpu Hospital, Tongji University School of Medicine, Tengyue Road 450#, Shanghai, 200090, China

## Abstract

The present study tested whether the LPS/TLR4 signal pathway in endometrial stromal cells is essential for the pathogenesis of adenomyosis. We tested the expression of TLR4, MD2 in the endometrium without adenomyosis (CE), the eutopic endometrium with adenomyosis (EuE) and the ectopic endometrium with adenomyosis (EE). We isolated the stromal cells from CE, EuE and EE (CESC, EuESC, EESC), treated with lipopolysaccharide (LPS) and TLR4 antagonist and detected the cell viability. And we also measured the key protein of the TLR4 signal pathway and inflammatory proliferation and invasive growth of experimental cells. We found that the viability of experimental cells treated with LPS was significantly greater than that of the non-treated cells, blocked by the TLR4 antagonist VIPER. TLR4 signal pathway and inflammatory proliferation and invasive growth of experimental cells stimulated by LPS, and it was inhibited by VIPER. This study suggested that stromal cells were activated by the TLR4 signalling pathway, which processed the cellular inflammatory proliferation and invasive growth involved in the pathogenesis of adenomyosis.

Adenomyosis is a common chronic gynaecological disorder associated with menorrhagia, dysmenorrhea and infertility^1^,^2^,^3^,^4^. As a special form of endometriosis, it is defined by the intramyometrial presence of glands and stroma derived from the basalis layer of the endometrium surrounded by the reactive hyperplastic or hypertrophic myometrium, causing chronic inflammation in the endometrium^5^,^6^,^7^,^8^. The exact pathogenesis of adenomyosis is still unclear. According to the published literature, there are different mechanisms of the pathogenesis of adenomyosis, such as *de novo* metaplasia of Mullerian remnants, invagination through the vascular or lymphatic channel^9^, mechanical tissue injury or stress reaction at the endo-myometrial interface, and downgrowth and invagination of the basalis layer of the endometrium into the myometrium^10^.

Emerging evidence suggests that this disease may be linked to the expression of inflammatory mediators and the induction of an immune response^11^,^12^,^13^,^14^,^15^. A few studies have found that the bacterial contamination of the uterine wall could be involved in the development of endometriosis/adenomyosis. Bailey *et al.* reported that endometriosis was associated with an altered profile of intestinal microflora in rhesus monkeys and that endometriosis was linked to higher concentrations of gram-negative bacteria. Kodati *et al.* showed genetic sequences homologous to shigella bacteria in the ectopic endometriotic tissue and proposed that shigella or shigella-like organisms may be the trigger for the immunological changes in the pelvic peritoneum that initiate the etiopathogenesis of endometriosis^16^. Additionally, studies suggest that compared with control women, higher colony formation of Escherichia coli in menstrual blood and endotoxin levels in menstrual fluid and peritoneal fluid of women with endometriosis^17^. The author even proposed the novel concept of a ‘bacterial contamination hypothesis for the development of endometriosis via the LPS/TLR4-mediated engagement of the innate immune response^17^.

The lower genital tract of humans is constantly exposed to various microorganisms, which could infect the upper genital tract through direct migration^16^,^18^,^19^,^20^,^21^. Sexually transmitted infections are common in domestic animals and humans^17^,^20^,^22^. In humans, sexually transmitted infections remain a major issue globally, and it is estimated that there are 340 million new infections each year, including Herpes simplex virus type 2, Chlamydia trachomatis, group B streptococcus, and acquired immunodeficiency syndrome (AIDS)^23^. Intrauterine infusion of bacteria or their pathogen-associated molecules (PAMPs) such as lipopolysaccharide (LPS), which is the major structural component of the cell wall of Gram-negative bacteria but is also the endotoxin responsible for much of the inflammation and shock associated with bacterial infection^24^, detected by pattern recognition receptors such as the Toll-like Receptors (TLRs)^25^. In addition to initiating inflammatory responses, TLRs have been shown to directly regulate cell proliferation and survival in a variety of biological settings^26^. Among these receptors, TLR4 was the first mammalian TLR to be identified by its role in LPS-responsiveness and based on its homology to Toll. TLR4-mediated production of different cytokines and growth factors and endometrial cell proliferation in response to LPS has been demonstrated previously^27^,^28^. However, little is known about the events that lead to the adhesion, growth and progression of endometrial cells that ultimately result in adenomyosis.

This study aimed to analyse the role of the TLR4 signalling pathway on the endometrial stromal cells and reveal the mechanisms of adenomyosis, providing a theoretical basis for clinical treatment.

## Materials and Methods

### Reagents

Antibodies for TLR4, MD2, Myd88, IL-6, TGF-β, VEGF, EGF, and MMP2 were purchased from Abcam (Cambridge, MA, USA). Antibodies for NF-κB p65 and glyceraldehyde-3-phosphate dehydrogenase (GAPDH) were purchased from Cell Signaling Technologies (Danvers, MA, USA). Secondary antibodies of goat anti-mouse FITC, goat anti-rabbit HRP and goat anti-mouse HRP were purchased from the Beyotime Institute of Technology, China.

### Subjects

A total of ten women between 20 and 38 years of age undergoing laparoscopy for pelvic pain, dysmenorrhea and/or infertility were recruited in this study. Neither the study group nor the adenomyosis-free group had been on hormonal medication in the last 3 months. All control women and women with adenomyosis had regular menstrual cycles (28–32 days). The exclusion criteria included patients who were subsequently diagnosed with uterine fibroid and patients with a history of coronary artery disease, hypertension, or hematologic disorders. The phase of the menstrual cycle was the early proliferative phase. The adenomyosis lesions were diagnosed by their macroscopic appearance according to the published criteria and the final diagnosis was mainly on histological examination of biopsy. Under strict asepsis, fresh tissue specimens were collected either in phosphate-buffered saline (PBS) buffer (100 U/mL penicillin, 100 μg/mL streptomycin added), stored at 4 °C, and processed within 2–18 h, or frozen on liquid nitrogen and stored at −80 °C for RNA and protein extraction.

### Ethics statement

The study was approved by the Research Ethics Committee of Chage to Yangpu Hospital, Tongji University School of Medicine, and in accordance with the tenets and guidelines of the Declaration of Helsinki. Written informed consent was obtained from all participants.

### Isolation of stromal cells in primary culture

Stromal cells were collected from the biopsy specimens of the eutopic and ectopic endometria derived from six women with adenomyosis and eutopic endometria without adenomyosis, respectively. The detailed procedures for the isolation of stroma were described previously^29^,^30^,^31^,^32^.

The characteristics of the cultured stromal cells were determined by morphological and immunocytochemical studies. Stromal cells exhibited flat, spindle-shaped, fibroblastic-like morphology and stained against vimentin. The endometrial stromal cell (ESCs) expressed vimentin which is a stromal cell cytoskeletal marker. An aliquot of stromal cells was placed in a two-chamber slide (Nunc) for immunostaining and the rest was used for culture. After 24 h, the slides were washed in PBS, fixed with 4% paraformaldehyde for 10 min and rinsed with PBS. The slides then were incubated in 0.1% Triton X-100 for 5 min and were incubated with human cytokeratin antibodies (mAb) (epithelial cell-specific) at a dilution of 1:50 (MNF 116; Dako, Denmark) or human vimentin mAb (stromal cell specific) at a dilution of 1:20 (V9; Dako) for 3 h at 37 °C. Immunocytochemical (IHC) staining was performed on at least three different isolated cells with similar results. The purity of stromal preparation was judged by positive cellular staining for vimentin.

### Immunohistochemistry assay in stromal cells

Formalin-fixed paraffin sections were deparaffinized in xylene and rehydrated through an ethanol series. The sections were then immersed in 0.3% hydrogen peroxidase for 15 min to deplete endogenous peroxidase and exposed to high pressure to activate their antigenicity. Sections were then incubated with blocking solution (PBS, 3% of BSA, 0.1% Triton-X 100) at room temperature for 30 minutes. The primary antibody of TLR4 (1:20, ab22048; Abcam), MD2 (ab24182; Abcam), Myd88 (ab2068; Abcam), NF-κB (ab7970; Abcam), IL-6 (ab6672; Abcam), TGF-β (ab66043; Abcam), VEGF (ab1316; Abcam), EGF (ab115562; Abcam), and MMP2 (ab86607; Abcam) were added and incubated at 4 °C overnight. Sections were washed with PBS, incubated with FITC–conjugated goat anti-mouse secondary antibody at room temperature for 1 hour (1:400), and washed three times with PBS at room temperature for 10 minutes. Images were obtained with a fluorescence microscope (OLYMPUS DP70; Olympus Co., Tokyo, Japan).

### Immunofluorescence assay

The cells were fixed with 4% paraformaldehyde at room temperature for 15 minutes and washed 3 times with phosphate buffered saline (PBS) at room temperature for 10 minutes and incubated with blocking solution (PBS, 3% of BSA, 0.1% Triton-X 100) at room temperature for 30 minutes. Primary anti-TLR4 (1:200, ab22048; Abcam) or anti-vimentin antibody (1:100, 5741S; CST) in primary antibody diluent (PBS, 3% BSA, 0.1% Triton-X100) was added and incubated at 4°C overnight; cells were washed with PBS, incubated with secondary antibody at room temperature for 1 hour (goat anti-rabbit the Alexa Fluor 555 and goat anti-mouse FITC), and washed 3 times with PBS at room temperature for 10 minutes. Images were obtained with a fluorescence microscope (DP70; Olympus).

### Treatment of stromal cells

The isolated stromal cells were cultured in triplicate (10^5^ cells /well) for 24 h to assess the basal (constitutive) production of cytokines. To evaluate the stimulated (induced) secretion of cytokines, after initial culture with serum containing RPMI medium, stromal cells were treated with lipopolysaccharide (LPS, 100 ng/ml) derived from Escherichia coli (serotype 0111:b4; Sigma, St Louis, MO, USA) for 24 hours. A blocking experiment was performed with anti-TLR4 antibody (500 nM VIPER) (Imgenex, USA) and LPS (100 ng/ml) in order to examine any change in the secretion of cytokines and growth factors in culture media with TLR4 antagonist antibody. After 24 h, the cultured media were collected in triplicate, pooled and frozen at −70 °C until testing.

### Detection of cell viability by CCK-8

Stromal cells (5.0 × 10^3^/well) were plated and treated in 96-well plates (three wells per group) with LPS (100 ng/ml) and LPS (100 ng/ml) plus VIPER (500 nM) for 24 h and 48 h, respectively. Approximately 10 μL of CCK-8 (Dojindo China CO., Ltd) was added to the cells, and the OD value of the cells was measured at 450 nm using an ELISA reader (bioswamp) according to the manufacturer’s instructions.

### Western blot analysis

Protein was extracted with radio-immunoprecipitation assay (RIPA) buffer containing a protease inhibitor cocktail and centrifuged at 12,000 × g for 15 minutes at 4 °C. The supernatant protein was quantified by bicinchoninic acid assay (BCA, Thermo Fisher Scientific, Rockford, USA) and stored at −80 °C. Total lysates were resolved in SDS–PAGE. Proteins were blotted onto a nitrocellulose membrane and incubated with primary antibodies and the corresponding secondary antibodies. Immune complexes were visualized by the use of an enhanced chemiluminescence western blotting system (BioRad, Richmond, CA).

### Real-time polymerase chain reaction

Real-time polymerase chain reaction (qRT-PCR) for TLR4 (tissue and cultured stromal cells) and MD2, Myd88, NF-κB, IL-6, TGF-β, VEGF, EGF, MMP2 (cultured stromal cells) derived from women with or without adenomyosis was performed with SYBR-Green Master Mix on an ABI 7300/7500 platform and related software as previously described (Applied Biosystems, Foster City, CA)(39). GAPDH was used as an internal control. The following primer sets were used: TLR4, forward primer: 5′ CCGCTTTCACTTCCTCTCAC 3′, reverse primer: 5′ CATCCTGGCATCATCCTCAC 3′; EGF, forward primer: 5′ GAAACTGTTGGGAGAGGAATCG 3′, reverse primer: 5′ AGCAAGGCAAAGGCTTAGAG 3′; Myd88, forward primer: 5′ CTGCCTCCTCCTTTCGTTGTAG 3′, reverse primer: 5′ GCTCTGCTGGTCCTTCTTAGTC 3′; TGF-β, forward primer: 5′ GACTACTACGCCAAGGAGGTC 3′, reverse primer: 5′ GAGAGCAACACGGGTTCAG 3′; VEGF, forward primer 5′ GACAGATCACAGGTACAG 3′, reverse primer: 5′ GAAGCAGGTGAGAGTAAG 3′; MMP2, forward primer: 5′ TTGACGGTAAGGACGGACTC 3′, reverse primer: 5′ GGCGTTCCCATACTTCACAC 3′; NF-κB, forward primer: 5′ GAATGGCTCGTCTGTAGTG 3′, reverse primer: 5′ TGGTATCTGTGCTCCTCTC 3′; MD2, forward primer: 5′ TATTGGGTCTGCAACTCATC 3′, reverse primer: 5′ GATCCTCGGCAAATAACTTC 3′; IL-6, forward primer: 5′ GCACCTCAGATTGTTGTTG 3′, reverse primer: 5′ AGTGTCCTAACGCTCATAC 3′; GAPDH, forward primer: 5′ CACCCACTCCTCCACCTTTG 3′, reverse primer: 5′ CCACCACCCTGTTGCTGTAG 3′. RNA (tissue) was extracted from frozen tissue samples using Trizol reagent. Stromal cells (5.0 × 10^5^/well) were plated and treated in 6-well plates (three wells per group) after 24 h with LPS (100 ng/ml) for 48 h. The RNA extraction of stromal cells was performed according to the Trizol manufacturer’s protocol. Complementary DNA was synthesized by reverse transcriptase at 42 °C for 1 hour and 75 °C for 5 minutes. The PCR cycling conditions were as follows: 94 °C for 7 min, followed by 40 cycles of 15 s at 94 °C and 60 °C for 45 s. The comparative Ct method was used and the amount of target was normalized to the GAPDH control (2−ΔΔCt) assuming an efficiency of 2.

### Enzyme-linked immunosorbent assay (ELISA) assay

Cell culture supernatants were collected for the detection of the concentration of TGF-β and IL-6 by a commercial ELISA kit (Bioswamp). A microplate reader (Labsystems Multiskan MS) was used to detect the absorbance at 450 nm of each well to compare experimental groups.

### Statistical analysis

The clinical characteristics of the subjects were evaluated by one-way analysis of variance. The data are expressed either as mean + SEM or mean + SD. The comparison of distributions of continuous variables between two or more groups was made using the Wilcoxon’s test, and Student’s t-test was used when the before–after comparison was made for the same samples. Analysis of variance was used to compare the differences in the mean among the groups when the data approximately represented a normal distribution. A P value of ≤0.05 was considered significant.

## Results

### Tissue levels of TLR4 in eutopic and ectopic endometria

**Figure 1:**
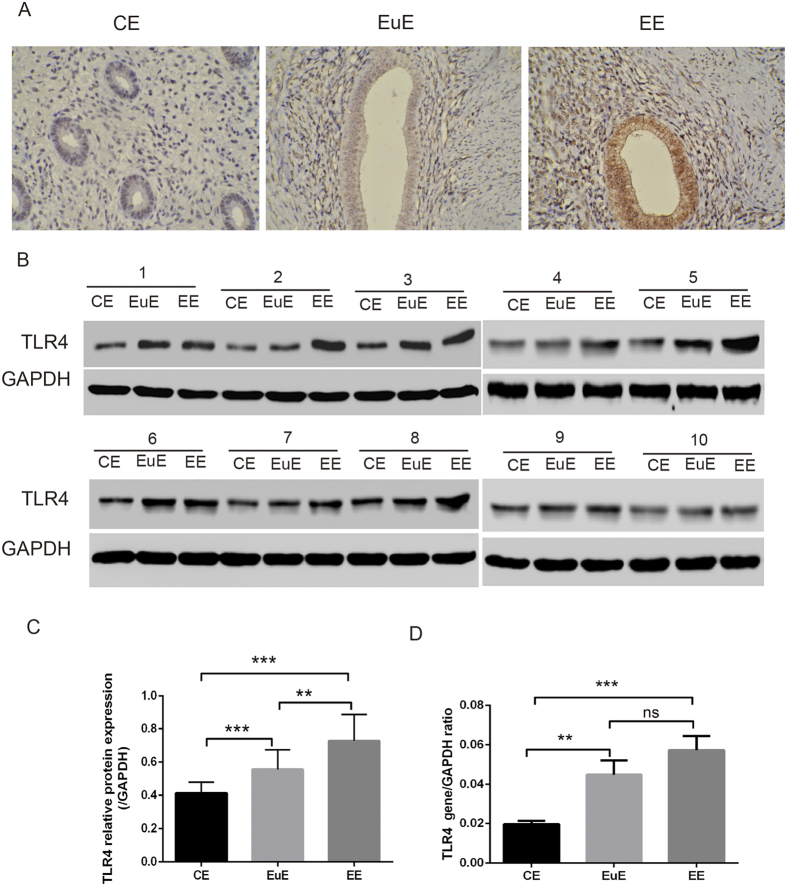
The expression of TLR4 in CE, EuE, and EE by IHC (**A**), WB (**B,C**), and RT-PCR (**D**). The gels had been run under the same experimental conditions. CE = endometrium without adenomyosis, EuE = eutopic endometrium with adenomyosis, EE = ectopic endometrium with adenomyosis. 1–10 means the sample label. (Ns p > 0.05, *p < 0.05, **p < 0.01, ***p < 0.005).

We detected both protein and gene expression of TLR4 in eutopic and ectopic endometrial tissue derived from women with adenomyosis and endometrium without adenomyosis. TLR4 was immunolocalized (Fig. 1A) in CE, EuE and EE. TLR4’s molecular weight of 78 kDa was also visualized by western blot analysis in CE, EuE and EE (Fig. 1B,C). This was confirmed at the mRNA levels (406 bp) in CE, EuE and EE (Fig. 1D). Although an apparent increase in the amount of TLR4 mRNA was found in EuE and EE, there was no significant difference in TLR4 expression between EuE and EE. The protein and gene expression of TLR4 in endometrial cells were reported elsewhere.

### Immunohistochemistry assay of isolated stromal cells

**Figure 2:**
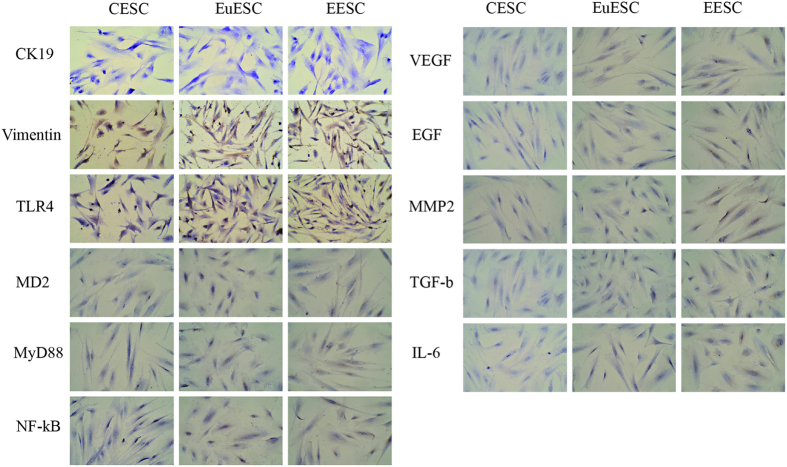
CESC, EuESC, and EESC were isolated. The immunoexpression of TLR4 was found in CK19-negative and vimentin-positive stromal cells. The expression of MD2, MyD88, NF-κB, VEGF, EGF, MMP2, IL-6 and TGF-β in CESC ranged 0–100, in EuESC range 200–210, and in EESC ranged 240–270 (×200).

**Figure 3:**
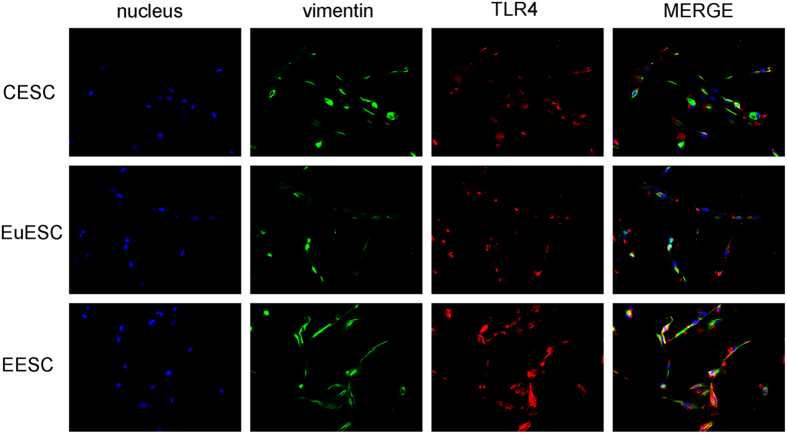
Immunofluorescence showed the expression of TLR4 in vimentin-positive stromal cells (×200).

TLR4 was immunolocalized in vimentin-positive and CK19-negative stromal cells, MD2, MyD88, NF-κB, VEGF, EGF, MMP2, IL-6 and TGF-β in CESC, EuESC and EESC were detected by IHC. Based on four-tiered (0, 1 + , 2 + , and 3 + ), or semiquantitative continuous variable as for the H score ((% at 0) × 0 + (% at 1 + ) × 1 + (% at 2 + ) × 2 + (% at 3 + ) × 3; range = 0 to 300) results^33^, the expression of molecules in CESC ranged 0-100, in EuESC range 200–210 and in EESC ranged 240–270 (Fig. 2). Tumors with scores 0 and 1 + were considered negative; 2 + was considered equivocal and required FISH reflex testing; 3 + was considered positive and eligible for trastuzumab^34^. Final magnification was × 200 using a light microscope. Double IF staining is to show TLR4 expression in vimentin-positive stromal cells (Fig. 3).

### LPS induced the viability of isolated stromal cells

**Figure 4:**
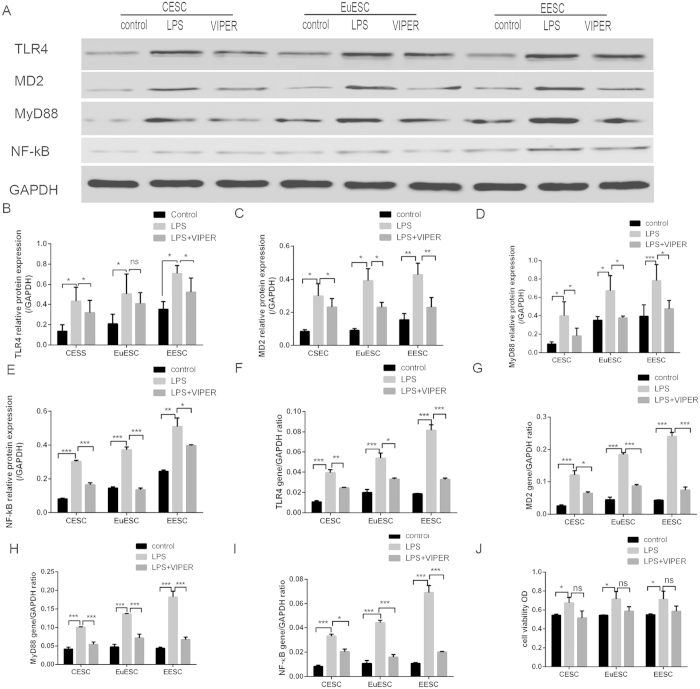
The cell viability was assessed by CCK-8 assay (J). The expression of TLR4, MD2, MyD88 and NF-κB in CESC, EuESC and EESC was measured by WB (**A–E**) and RT-PCR (**F–I**) after treated with LPS and VIPER. CESC: stromal cells from endometrium without adenomyosis, EuESC: stromal cells from eutopic endometrium with adenomyosis, EESC: stromal cells from ectopic endometrium with adenomyosis (Ns p > 0.05, *p < 0.05, **p < 0.01, ***p < 0.005).

In our initial time-dependent and dose-dependent study, we found a maximum increase in the levels of cell growth at 24–48 h and in response to 10–100 ng/ml of LPS. Therefore, we obtained the viability of stromal cells in response to 100 ng/ml of LPS with a treatment duration of 24 h by CCK-8 assay. We found that the viability of stromal cells was significantly higher in the culture media of LPS-treated (100 ng/ml) cells than at other times and doses (Fig. 4J). Anti-TLR4 (VIPER) decreased the effect, but with no significance.

### LPS induced the expression of TLR4, MD2, Myd88, and NF-κB in isolated stromal cells

Our previous study demonstrated that LPS could stimulate stromal cell viability. As an initial inflammatory mediator, bacterial endotoxin or lipopolysaccharide (LPS) has been recently reported to regulate the TLR4-mediated growth of endometriotic cells. The appropriate concentration of LPS was determined to be 100 ng/ml by our preliminary experiment (data not shown). Western blotting (Fig. 4A–E) showed that the expression of TLR4, MD2, MyD88 and NF-κB in CESC, EuESC and EESC was significantly higher after LPS treatment, and VIPER significantly decreased the effect. We have done statistical analysis among the group that there are no different pattern among the three groups. As shown by real-time PCR (Fig. 4F–I).

### LPS induced the expression of IL-6, TGF-β, VEGF, EGF and MMP2 expression in isolated stromal cells

**Figure 5:**
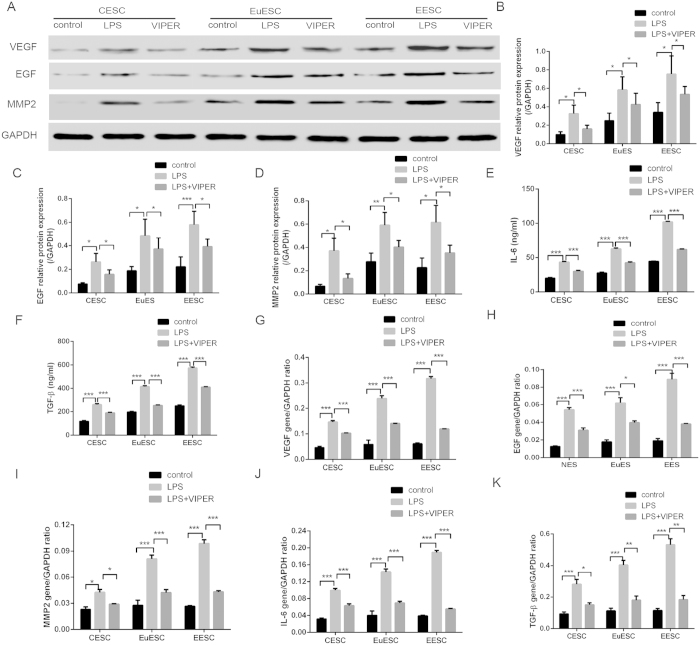
The expression of VEGF, EGF, MMP2, IL-6, TGF-β in CESC, EuESC and EESC was measured by WB/ELISA (**A–F**) and RT-PCR (**G–K**) after treated with LPS and VIPER. (Ns p > 0.05, *p < 0.05, **p < 0.01, ***p < 0.005).

We found that the TLR4 signal pathway played an important role in the pathogenesis of adenomyosis. TGF-β and IL-6, which play important roles in the process of infiltration and proliferation of stromal cells, were detected by ELISA assay (Fig. 5E,F). WB showed that the protein expression of VEGF, EGF and MMP2 increased significantly in CESC, EuESC and EESC after treatment with LPS for 24 hours (Fig. 5A–D). RT-PCR showed that the expression of VEGF, EGF, MMP2, TGF-β and IL-6 was increased significantly in CESC, EuESC and EESC after treatment with LPS for 24 hours (Fig. 5G–K). VIPER antagonized the effect. These results suggested that LPS could stimulate stromal cells and promote the expression of the infiltrative and proliferative factors in adenomyosis.

## Discussion

The role of TLRs as a first line of defence against microbial infection has been well established^14^,^15^,^35^,^36^,^37^. The potent inflammatory response induced by TLRs is protective in most cases, as the pathogens are destroyed before they can harm the host. However, if inflammation persists, it can result in autoimmune/inflammatory diseases^26^,^38^,^39^,^40^,^41^. Therefore, TLR signalling needs to be tightly regulated^42^,^43^. This is achieved through negative feedback loops that are present in most cells, as well as tissue-specific signalling mechanisms. The endometrium requires specific regulatory mechanisms due to constant exposure to microbes and their products and the damage caused by inflammation as a result of infection.

In this study, we first analysed the expression of TLR4 in EuE, EE and CE and found that TLR4 was higher in EuE than CE and was the highest in EE. Similar results were also reported recently by Allhorn *et al.*, who demonstrated that ectopic endometriotic lesions showed a significant increase in TLR4 mRNA expression compared with corresponding eutopic tissue, providing a direct basis for uterine infection, which could be a local biological event in the development of adenomyosis^44^. Besides LPS, there are a number of other exogenous and endogenous ligands for TLR4. Peter *et al.* reported that human papillomaviruses(HPV) were detected in 11.3% of endometriosis lesions, corresponding to 13.2% of patients. In addition, 27.5% of control tissues were positive for HPV, presenting a medium to high risk^45^. The author reasoned initially that a possible viral or chlamydia infection of the endometrium along with retrograde menstruation could enhance invasiveness of endometrial cells in the intraabdominal or the myometrial region. Whether infection is responsible for the development of adenomyosis has been the focus of recent research. Our findings proposed that adenomyosis may be associated with intrauterine infection.

Further, we isolated eutopic and ectopic endometrial stromal cells from patients with adenomyosis and endometrial stromal cells without adenomyosis. The purity of isolated primary stromal cells was crucial to our study. Delbandi *et al.* clearly showed that isolated ESCs from all three sources expressed vimentin and nestin, but failed to express the epithelial marker cytokeratin^46^. Here, most stromal cells were found to specifically express vimentin, which indicated that the ESCs were successfully isolated.

We then tested the expression of the TLR4 signal pathway molecule (TLR4, MD2, MyD88 and NF-κB) following LPS exposure in ESCs by RT-PCR and western blotting. Our results showed that TLR4, MD2, MyD88 and NF-κB significantly increased after LPS treatment. Our current findings indicated that TLR4-MyD88-NF-κB had vital functions in the development of adenomyosis. Another study revealed that bacterial microflora and Toll-like receptor 4 (TLR4), but not TLR2, functioning via the MyD88-NF-κB-dependent pathway was required for hepatic fibrogenesis^47^. Reinaldo *et al.* reported that dysregulation of NF-κB activation in the endometrium of endometriosis patients may explain some of the biological alterations associated with endometriosis^48^. Scientific evidence strongly suggests that NF-κB activity in endometriotic cells stimulates inflammation and cell proliferation and inhibits apoptosis, favouring the development and maintenance of adenomyosis.

Adenomyosis is associated with the increased secretion of pro-inflammatory cytokines, impaired cell-mediated immunity and neo-angiogenesis^49^. To date, many cytokines suspected to be involved in adenomyosis have been analysed. In this review, we concentrated on VEGF, EGF, MMP2, IL-6, and TGF-β because we suspect that they may play a major role in the biological processes leading to the establishment and maintenance of adenomyosis. This study clearly showed that VEGF, EGF, MMP2, TGF-β, and IL-6 were significantly higher in CESE, EuESC and EESC after LPS treatment. Recent studies revealed that the number of activated macrophages increases in the peritoneal fluid (PF) of patients with endometriosis. Peritoneal macrophages synthesize and secrete different cytokines, such as IL-6, IL-8, IL-10, tumour necrosis factor (TNF)-a, and TGF-β, which may facilitate the adhesion, invasion, or proliferation of endometrial cells and the progression of endometriosis^49^,^50^. Among others, IL-6 and IL-8 have been studied extensively and implicated in the pathogenesis of endometriosis. IL-6 is a pro-inflammatory cytokine involved in the activation of T cells; it also promotes the differentiation of B cells. A separate study used a much lower threshold of 1.3 pg/ml and yielded a sensitivity of 81% with a specificity of only 51% to diagnose all women with endometriosis, regardless of stage^51^. TGF-β is implicated in gene expression, cell motility, proliferation, apoptosis, differentiation, immune responses and tumorigenesis^52^,^53^. Cell invasion is a central element of tumour formation and progression. It is regulated both by the engagement of adhesion receptors with proteins of the extracellular matrix (ECM) and by the secretion of collagenolytic enzymes, called matrix metalloproteinases (MMPs)^54^. Accordingly, under the conditions of the inflammatory microenvironment, the ectopic endometrial tissue of adenomyosis may also be highly invasive, which facilitates the extension of the basalis layer of the endometrium into the myometrium.

Besides, endometriosis/adenomyosis is an estrogen-dependent chronic inflammatory disease mostly affecting women of reproductive age. The development and regression of adenomyosis foci are associated with the level of estrogen^55^. Pain levels and uterine innervation of patients with adenomyosis are also regulated by estrogen^56^,^57^. The level of estrogen secreted by the ovary was similar among adenomyosis, endometriosis, and normal individuals. However, the uteri local estrogen levels were higher in patients with adenomyosis and endometriosis than in the controls because of the increased synthesis of aromatase and 17b-hydroxy cholesterol dehydrogenase (HSD). Estrogen synthesized from androstenedione by the effects of aromatase and 17b-HSD could accelerate adenomyosis and endometriosis by promoting the growth of ectopic endometrium^57^. From the results herein, we believe that locally increased estrogen levels and inflammatory status may be responsible for adenomyosis.

In this study, reducing estrogen interference, we choose the patients in early proliferative phase to discuss the LPS/TLR4-induced signal pathway in eutopic and ectopic endometrium and its possible association with reproductive outcome in women with adenomyosis. Current on-going studies from our laboratory address the relationships between infection and stromal cells’ acquisition of the invasive phenotype, and we speculate that TLR4-dependent signalling activated by endogenous ligands promoted the secretion of different cytokines and growth factors, stimulated endometrial cell proliferation, recruited and activated immune cells (macrophages, DCs, NK cells), triggered the local inflammatory response, activated the fabrication and maintenance of an inflammatory microenvironment by the specific immune system, further induced stromal cell proliferation and invasion, amplified the signalling effect of the local inflammatory reaction, and eventually led to the development of adenomyosis. Our current findings may provide clues in targeting TLR4 in new therapeutic strategies for women with adenomyosis.

## Additional Information

**How to cite this article**: Guo, J. *et al.* LPS/TLR4-mediated stromal cells acquire an invasive phenotype and are implicated in the pathogenesis of adenomyosis. *Sci. Rep.* **6**, 21416; doi: 10.1038/srep21416 (2016).
